# Gait Analysis Using Animal Models of Peripheral Nerve and Spinal Cord Injuries

**DOI:** 10.3390/biomedicines9081050

**Published:** 2021-08-19

**Authors:** Gheorghita Isvoranu, Emilia Manole, Monica Neagu

**Affiliations:** 1 Husbandry Unit, Victor Babes National Institute of Pathology, 99-101 Splaiul Independentei, 050096 Bucharest, Romania; gina_isvoranu@yahoo.com; 2 Laboratory of Cellular Biology, Neuroscience and Experimental Myology, Victor Babes National Institute of Pathology, 99-101 Splaiul Independentei, 050096 Bucharest, Romania; 3 Pathology Department, Colentina University Hospital, 19-21 Sos. Stefan cel Mare, 020125 Bucharest, Romania; neagu.monica@gmail.com; 4 Immunology Laboratory, Victor Babes National Institute of Pathology, 99-101 Splaiul Independentei, 050096 Bucharest, Romania; 5 Doctoral School of Biology, Faculty of Biology, University of Bucharest, 91-93 Splaiul Independentei, 050095 Bucharest, Romania

**Keywords:** gait analysis, animal models, nerve injuries, regeneration, CatWalk

## Abstract

The present review discusses recent data regarding rodent models of spinal cord and peripheral nerve injuries in terms of gait analysis using the CatWalk system (CW), an automated and exceptionally reliable system for assessing gait abnormalities and motor coordination. CW is a good tool for both studying improvements in the walking of animals after suffering a peripheral nerve and spinal cord lesion and to select the best therapies and procedures after tissue destruction, given that it provides objective and quantifiable data. Most studies using CW for gait analysis that were published in recent years focus on injuries inflicted in the peripheral nerve, spinal cord, and brain. CW has been used in the assessment of rodent motor function through high-resolution videos, whereby specialized software was used to measure several aspects of the animal’s gait, and the main characteristics of the automated system are presented here. CW was developed to assess footfall and gait changes, and it can calculate many parameters based on footprints and time. However, given the multitude of parameters, it is necessary to evaluate which are the most important under the employed experimental circumstances. By selecting appropriate animal models and evaluating peripheral nerve and spinal cord lesion regeneration using standardized methods, suggestions for new therapies can be provided, which represents the translation of this methodology into clinical application.

## 1. Introduction

Most neurological conditions, such as central and peripheral nervous system damage, are accompanied by gait changes. It is commonly agreed upon that the details of animal limb movements during locomotion, whether in injury or during recovery from spinal cord or peripheral nerve lesions, determined using appropriate models, are of utmost importance for the assessment of such conditions. Several functional tests are used to evaluate the motor recovery after experimental nerve injury and to appraise the potential regenerative therapies. The option for certain tests depends both on the lesion model and on the costs, since specific commercial systems have higher costs.

In their review of the many works on spinal cord injury, Vogelaar and Estrada [1] showed that the most popular methods for gait evaluation are the Basso, Beattie, and Bresnahan (BBB) locomotor score and subscore (for rat) and the Basso Mouse Scale (BMS, for mouse) determination, horizontal ladder rung test and gridwalk, automated gait analysis methods, MotoRater and kinematic analysis, sensory tests, and forelimb tests. Of these methods, the automated gait analysis methods have been increasingly utilized in recent years, based on the volume of scientific works describing their use. The simple visualization of the animal walk, performed by a researcher, or the footprint measurements made from markers fixed over chosen articulations, are less commonly used since they are subject to multiple errors. Thus, increasingly sophisticated analysis tools have been developed to observe that which cannot be observed with the naked eye, to reduce the time dedicated to empirical measurements, and to avoid animal stress.

Gillis et al. conducted 2D analysis studies of joint kinematics in rats using two high-speed video cameras positioned perpendicular to the direction of movement (sagittal plane), in which the rat hind-limb movement was assumed to be planar [2]. However, this method ignores the small joint movements in the transverse plane, which are relevant in injury model analysis. More advanced, a 3D analysis system was developed to measure three-dimensional joint kinematics in animal models, allowing subjects to move naturally [3]. The system simultaneously used multiple cameras, allowing for a precise measurement of each marker (reflective markers attached to bony landmarks on the overlying skin) position in 3D space. However, the movement of skin relative to the bony landmark resulted in artefactual errors in motion measurements.

Eftaxiopoulou T et al. in 2014, investigated a method for detecting subtle locomotor changes in rat gait due to sciatic nerve injury induced by injecting a transient nerve block in the left hind limb. They used a combination of an optical motion tracking system and DigiGait™, an automated gait analysis system, simultaneously putting together the dynamic and kinematic parameters. Therefore, a more robust method for the quantification of gait changes in rats was established. The most important parameters measured using the DigiGait system were stance and swing time, stride length, and paw contact area. However, this method did not provide a quantitative measure of joint kinematics, thus lacking spatiotemporal parameters [4]. DigiGait uses a motorized, transparent treadmill belt, and is a predecessor of CatWalk technology.

In the last 3 years, over 100 papers have described gait analysis in rodent models using the CatWalk system (CW), with most studies focusing on injuries inflicted on the peripheral nerve, spinal cord, and brain. In general, the studies evaluate regeneration processes and analyze the effect of various drugs and procedures that enhance the recovery of nervous tissue.

**Figure 1:**
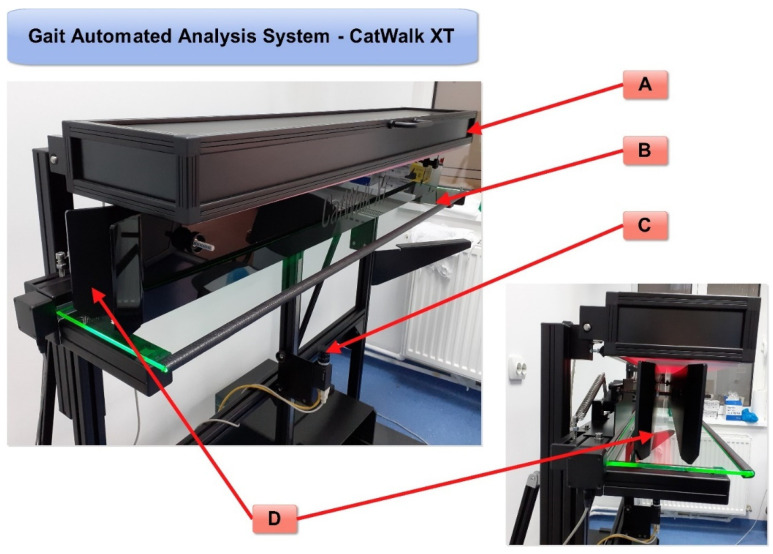
Automated gait analysis system—CatWalk XT: (**A**)—red LED lights in the ceiling; (**B**)—green LED lights along the walkway (reflected by the plantar surface of the rodent’s paw); (**C**)—a high-speed color camera for accurate spatial and temporal resolution; (**D**)—an adjustable corridor that directs the free movement of the rodents in a straight line with a glass walkway that enables CatWalk XT’s illuminated footprints technology (original photos).

In these analyses, the measurement of motor function before and after nerve injury in animal models is very important for the assessment of nerve regeneration. There are several methods for the evaluation of nerve regeneration, many of which are based on the visual observation of laboratory animals moving in an open field. Nevertheless, all these observation methods require the training of observers that can be prone to human error and subjectivity. The automated gait analysis system CatWalk XT (Figure 1) can overcome possible human errors of observation since specialized software is used to objectively measure several aspects of gait, and it was applied to assess rodent walk using high-resolution videos [5]. Older studies have used a CW system without automatic analysis of paw parameters; therefore, we wish to focus attention on the studies that have used a CW XT with an automatic analysis system for monitoring gait and motor functions.

Within this type of automated gait analysis, various parameters are registered and evaluated, as further presented. The experience gained using this analysis system would add information to the experimental models of neurological injuries and regeneration. In our review, we present the main features and methodology of this system, the main regeneration events that can be analyzed, and recent studies that have used this system for assessing peripheral nerve and spinal cord injuries.

## 2. Evaluation of Gait Analysis System: Parameters of Rodent Walking

Several approaches for gait analysis have been described using rodents in experimental models. Observational scoring is performed by introducing the animal in an open field and directly scoring or recording its movement by video. Rodent gait rapidity surpasses the human eye and, therefore, it is easier to assess subtle gait changes in video recording [6]. Other studies have used treadmills and food incentives, but a natural gait with its unique characteristics would give investigators optimal results [7]. As our experience is accumulating, we can ascertain that, before registering the natural gait of experiment animals, proper handling and allowing accommodation to the new environment are essential.

Basso, Beattie, and Bresnahan (BBB) scoring is a classical behavioral evaluation method in which the behavioral observation of a paralyzed rodent is conducted for 4 min in an open field (90 cm diameter). This type of observation is subjective and, therefore, prone to human error. Moreover, the studies on nonrodent models have demonstrated that a disruption of the dorsal pathways alone may not result in detectable and significant locomotion deficits [8]. Electroencephalography (EEG) and its various versions, such as electrocorticography (ECoG), somatosensory evoked potential (SSEP), and magnetoencephalography (MEG), have proven valuable in the evaluation and assessment of spinal cord injury [9]. SSEP is a more objective assessment method for longitudinal studies in spinal cord injury model [10]. SSEP is a tool that is used to assess the onset and progress of neuronal injuries, and it is sensitive enough for the detection of both endogenous recovery and the effectiveness of treatments. Compared with the BBB score, SSEP is a more direct method for the measurement of cortical projections of neuropathway functionality. If SSEP is used in longitudinal monitoring, the signal analysis is sensitive enough to recognize minute transient insult and minimal functional recovery post-spinal cord injury [11]. Recent studies have assessed BBB in comparison to SSEP and it was shown that the two methods complement each other and, therefore, both SSEP-based and BBB-based methods could be adopted for an improved evaluation of transection in spinal cord injury rodent models [10].

**Figure 2:**
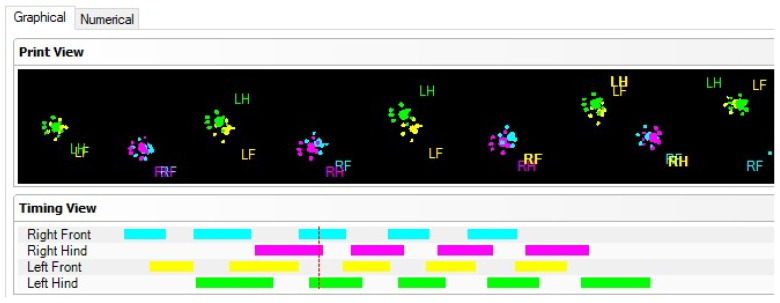
Primary data registered using CW system in normal speed passing of a Wistar rat. Upper panel depicts the paw imprints for each definition of right (R) and left (L) paw, and front (F) and hind (H) paw. Lower panel depicts the numerical automated registration after the paws are defined.

The CatWalk gait system consists of a glass walking platform illuminated with a green light, a high-speed digital camera, and software for recording, analyzing, and processing data obtained from rodent paw prints (Figure 1). The green light is reflected in the glass, except for the points that are touched, which are imprinted by the paws. The light is scattered and illuminates the contact area. The intensity of the lighting area, which is proportional to the pressure exerted by the footprints, is captured by the video camera and analyzed by the software. Data analysis requires the classification of footprints as right/left front and right/left hind paw (Figure 2). Below, we show some images of normal rats walking from our experimental data.

**Figure 3:**
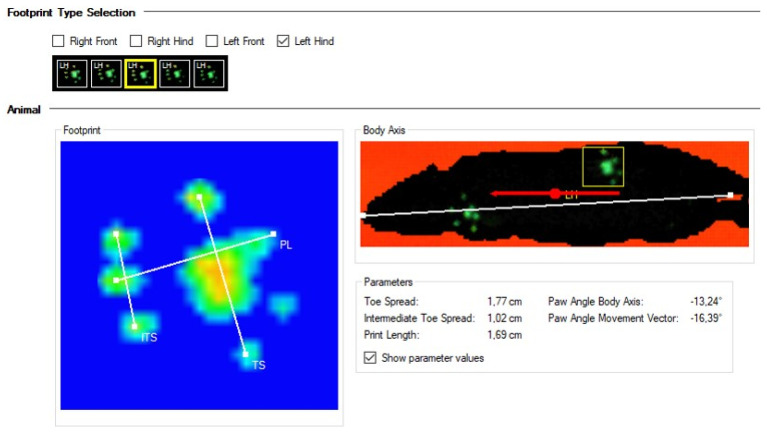
Individual paw evaluation in a Wistar rat registered using CW system at normal passing speed. Upper panel depicts the indicated paw print. Lower panel depicts graphical and numerical registration of the parameters (toe spread, intermediate toe spread, print length, paw angle body axis (paw angle versus body angle), and paw angle movement vector).

Although the contour of the body is visible on the CW images (Figure 3), it is not considered a print. Only the light footprints (in green) are calculated. Moreover, some parameters such as the run duration (s), average speed of run (cm/s), and maximum variation of speed (%) can be obtained without the selection and classification of paw prints.

The resulting CW data, using powerful software, can be transformed in a quantitative analysis of numerous parameters, but also a qualitative description of the gait.

The paw print classification results are expressed either graphically or numerically (Figure 3). A paw print measurement involves the evaluation of three parameters, including the print length (PL), toe spread (TS), and intermediate toe spread (ITS) (Figure 3). Paw print measurements provide data on the length of the print, the distance between the toes, and the position of the foot relative to the body of the animal.

**Figure 4:**
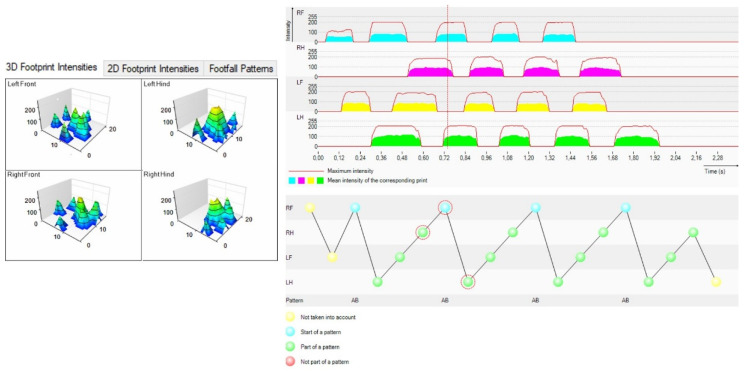
The 3D representations of all four paws in a Wistar rat registered using the CW system in normal speed passing. Left panel depicts 3D registration of each of the four paws. Right panel depicts the intensity of the print and the time periods for this intensity is sustained.

The software also enables the 3D and 2D visualization of paw prints, as well as the presentation of the step pattern (Figure 4).

The CW software automatically calculates a large number of parameters (Table 1), which can be divided into (1) parameters associated to individual paw prints: print size (length, width, and area), stand and swing duration (duration of posture and balance), maximum contact area, and average intensity (the intensity of paws); (2) parameters related to paw print positions, such as relative paw position, stride length, base of support; (3) parameters related to coordination: front–hind limb coordination (FL–HL coordination), phase dispersions, step pattern, and regularity index (RI). RI depends on the normal sequence of steps, when the animal walks on all four paws, one after the other, without interrupting this movement. The RI index of healthy animals is 100%. Regaining locomotor coordination is an important step in recovery from nerve damage, and the main condition for coordination is the communication between the different parts of the locomotor system [12].

The speed of locomotion is an extremely important parameter because it analyzes runs with a crossing time of between 1 and 2 s [20].

In a sciatic nerve crush experiment, Bozkurt et al. [20] analyzed the CW parameters and showed their dynamics in time.

An important parameter for the peripheral nerve study, which can be calculated using the software, is the sciatic functional index (SFI) used for studying the pathology and recovery of sciatic nerve injury. The calculation of SFI is based on the representation of the affected paw compared to the contralateral paw [20].

The main parameters analyzed using CW software in Bozkurt experiments and their description follow.

**The intensity of the hind paws** indicates the mean intensity of all the pixels that contribute to the maximum paw print area, and it is not considered a clear static parameter (such as individual paw parameters) nor a clear dynamic one (such as stance or swing phase parameters). This value could reflect the pressure exerted by the paws during gait. Thus, after sciatic nerve crushing, the intensity of the injured paw print was reduced to 50–60% of the pre-operative value [5,14], and at 2–3 weeks after lesion, this value was markedly reduced, reflecting a 100% recovery of this parameter.

**The individual paw parameters** related to paw print size, length, and width, as measured in the same experiment, showed a reduction to 10–15% after sciatic nerve crush, even at 12 weeks following injury [20].

**Dynamic paw parameters**, which have a temporal nature, were also analyzed.

One of these values is the stance phase of the paw ipsilateral to the sciatic nerve lesion. For example, in the sciatic nerve crush experiment, this parameter was reduced to about 50% of the pre-operative value for 3 weeks after injury [20], with recovery to the pre-operative values at a later time point. Another value to be considered is the swing phase that has an inverse effect compared with the stance phase in the sciatic nerve crush experiment. Duty cycle is a measure reflecting stance duration/sum of stance and swing duration. After sciatic nerve crush, this value showed a similar trend to that of stance duration [20]. The fact that the authors did not find a significant difference in the stride length of the hind paws at any time point after injury is due to the decreased speed at which the hind paws moved during swing, although the duration of the swing phase was increased.

**The coordination-related parameters**, as defined above, show the order of paw placements. Before starting any experiment, all the animals must be observed regarding the value of these parameters, specifying the order of the placement of the paws, i.e., the Alternate-b (Ab) type. In the case of sciatic nerve crush, no significant difference was noted (regularity index and NSSP), but the timing at which paws were placed after each other was altered [20]. This timing is quantified in the parameter named coupling measures or phase lags [21]. In the case of sural nerve injury, the most obvious changes were the coupling of the diagonal between the affected right hind paw and the left forepaw, and in the coupling of the girdle between the affected right hind paw and the left hind paw [20], reflecting a later positioning of the affected hind paw. The high variability of the affected ipsilateral coupling prevented the detection of strong statistically relevant effects.

In a spinal cord injury study, Timotius et al. [22] recently showed the importance of using a combination of CW parameters, which is superior to single parameter analysis. They combined nine of the most used parameters into a gait index score based on linear discriminant analysis (LDA) that is not affected by factors such as sex, size, and strain. It was a new and unique example of CW data analysis that included swing time (the duration of noncontact with the walkway of a specific paw), stride length (the distance between the center points of the two consecutive positions of the same paw), duty cycle (%) (stand time/step cycle), base of support (BOS) in cm (averaging the width on the *y*-axis between either forepaws or hind paws), regularity index (RI) (%) (the ratio of the number of normal step sequence patterns (NSSP) times four and the total number of paw placements), body speed (cm/s) (calculated from each paw by dividing the distance by the time of two consecutive initial contacts), sequence AB (%) (percentage of a specific LF–RH–RF–LH paw sequence, where LF is left forepaw, RH is right hind paw, RF is right forepaw, and LH is left hind paw), Max contact (%) (the ratio of “max contact at (s)” to the stand time (s), where “max contact at (s)” is the time when maximum contact with the walkway is measured—this is calculated from each paw and related to the point in which the braking phase turns into the propulsion phase).

It is important to mention that all the animals must be trained to move in the specially created corridor of the catwalk to ensure that at least three nonstop runs can be recorded for appropriate analysis. This training, ideally conducted by a restricted number of handlers, lasts at least a week.

## 3. Outlines of Peripheral Nerve and Spinal Cord Injury

Nerve injuries have been experimentally studied, especially in rodents, considering the relatively low cost of developing this type of animal model. The functional recovery evaluation upon regeneration is especially important and, hence, automated gait analysis can record many parameters such as paw print area, paw swing speed, and interlimb coordination. The digital data obtained within a CW system can illustrate the comparative evolution of animal walking before and after nerve injury, either in the central or peripheral nervous system, the locomotor behavior in time, and during regeneration. The spinal cord injuries can also be studied and evaluated with the aid of this system. As already stated, it is particularly important that the animals should be properly trained before experiments, and the determination of appropriate software settings through prior data acquisition should be thoroughly performed. There are also limitations in the application of this method, such as in the evaluation of sciatic nerve regeneration after neurotmesis due to limited functional recovery [23].

A spinal cord injury (SCI) leads to ascending and/or descending tract interruption, provoking paralysis, sensitivity loss, spasticity, and neuropathic pain. Even if the SCI does not directly affect the peripheral nervous system, the peripheral nerves are indirectly affected as a consequence of this injury, given that the cell bodies reside in the dorsal root ganglia and spinal cord. In turn, peripheral nerve injury (PNI), such as axotomy, can induce changes in the spinal cord, affecting neuron cell bodies. Thus, SPI and PNI, although they are independent phenomena, can also be seen as connected processes, whereby the degradation of one indirectly induces the degradation of the other. Moreover, the therapeutic methods applied for the regeneration of the peripheral nerve can induce positive effects in the partial recovery of motor neurons in the spinal cord.

Below, we will analyze the effects of SPI and PNI in animal models in some recent in vivo studies and the importance of behavioral assessments using CatWalk.

### 3.1. Spinal Cord Injury

An SCI is an incapacitating condition causing weakened motor function or even full paralysis, and there are more than 2.5 million people worldwide currently living with an SCI [24]. In the following period, after the initial mechanical injury to the spinal cord, there are clear processes in which inflammation and apoptosis induce additional injuries to the initially harmed tissues [25]. These processes markedly delay recovery [26]. An SCI is a complex process leading to massive necrosis and hemorrhage with extensive tissue destruction. Areas of necrosis turn into the cavity of injury where inflammation is localized and fluid accumulates, and they expand in time, during the long inflammatory phase. This cavity grows in reactive astrogliosis, while the severity of macrophage infiltration declines in the resolution phase. Necrosis occurs on the surface of the spinal cord, which becomes infiltrated by inflammatory cells, including macrophages, fibroblasts, and capillary blood vessels. Over time, the area is fibrosed and becomes a typical scar. The assessment of all these intimate processes requires a plethora of methods to indicate the presence of a regeneration process [25].

The key to approving new therapies and translating them into the clinic lies in the development of effective and appropriate animal models. Nevertheless, there are multiple factors that can influence this evaluation, such as species differences and having good predictive animal models and methodologies that cover relevant preclinical and clinical studies.

Therefore, a comparative neuroanatomical assay of the murine spinal cord, a nonhuman primate, and humans was performed [27]. Murine strain differences have also been studied in terms of SCI models, which were investigated in genetically different strains such as C57BL/6 and Swiss Webster mice. Besides the immunohistochemistry differences identified in the lesion site, behavioral assessments using CatWalk™ revealed significant differences. Hence, increased mobility (average speed) and a differential sensory response in Swiss Webster mice compared to C57BL/6 mice were observed. The behavioral differences correlated with the histological assessment of injury, which revealed a reduced microcavity formation and lesion volume in Swiss Webster mice [28].

A recent study has shown that besides mouse strain differences, there are differences due to age and, hence, this is also a factor that induces heterogeneity in SCI models. This finding can be reflected when testing various therapies. Therefore, C57Bl/6 and immunodeficient Rag2gamma(c)−/− and Agouti SCIDxRag2Gamma(c)−/− hybrid mouse strains were investigated. Differences were found in the post-SCI contralateral forepaw duty cycle and regularity index. Moreover, when investigating young (e.g., 3–4 months old) and aging (e.g., 16–17 months old) Rag2gamma(c)−/− mice, age-related pre-injury differences in strength and rearing were found. When testing therapeutic SCI approaches, the experimental design and analysis has to take into account all these differences [24].

The type of trauma to the spinal cord also affects walking function after injury. Guo et al. showed in a contusion/distraction experiment in rats that certain behavioral outcomes were linearly correlated with histological outcomes, and gait deficit and recovery may be affected by spinal cord trauma type [29]. The authors used CW for gait analysis of walking and the ladder rung walking test to quantify skilled locomotor movements. The paw support, inter paw coordination, front- and hind paw kinematics, and skilled movements were significantly different before and after injury. There is a clear differentiation between contusion and distraction, as CW parameters differed in correlation with the trauma type. Thus, at 2 weeks post-injury, step sequence duration, diagonal support, forelimb intensity, forelimb duty cycle, forelimb paw angle, and forelimb swing speed were greatly affected in distraction compared to the contusion experiment. In contrast, after 8 weeks post-injury, the hind limb stand was greatly affected in contusion compared to the distraction experiment. Other parameters returned to normal after 8 weeks post-contusion, such as diagonal coupling-variation, girdle coupling-variation, ipsilateral coupling-mean, forelimb maximum contact, forelimb intensity, forelimb paw angle, and number of forelimb misplacements. In post-distraction experiments, a number of other parameters returned to normal, such as the step sequence duration, ipsilateral coupling-variation, forelimb stand, forelimb duty cycle, hind limb swing duration, hind limb swing speed, and number of forelimb slips [29].

Several new therapeutically approaches are tested using animal models of SCI and hence, CatWalk equipment, such as neurotransplantation, cellular and molecular therapies.

Brain-derived neurotrophic factor (BDNF) is a secreted protein that can improve SCI, because it promotes neuron survival and synaptic plasticity. In a mouse model with SCI, a single dose of mRNA for BDNF incorporated in nanomicelles increased the factor secretion in the injured spinal cord and the motor functions registered with both Basso Mouse Scale and CatWalk Automated Gait Analysis System. The obtained results showed increased motor function recovery post-injury [26].

Another very recent therapeutic experiment using hyper-interleukin-6 delivery in C57BL/6;129/J-TgH (PTEN-flox) mice produced a functional recovery after a severe spinal cord crush. One of the analysis methods used for automated evaluation footprints, basal motor behavior, and coordination during locomotion was the CatWalk XT system. After 8 weeks post-injury, CW testing analyzed the following parameters: maximum area of the paw, total surfaced area of the complete footprint, stride length between the successive placements of the same paw, base support (average width between the footprints of the hind paws), and regularity index (coordination between hind and forelimb movement) [30]. Neurotransplantation, whether autologous or heterologous, is another therapeutic approach that was tested in animal models using the CW system.

An SCI causes severe loss of grey and white matter, inducing a motor and sensory functions deficit below the lesion. In a murine experimental model, cell transplantation of murine clonal embryonic neuroectodermal stem cells was investigated to reduce secondary damage. NE-GFP-4C stem cells cell line were used for cell transplantation in thoracic spinal cord contusion injury. Various functional tests were used, among which was CW evaluation. The stem cells that were transplanted were found integrated and differentiated (e.g., in neurons, astrocytes, and oligodendrocytes). The functional improvement tested with CW suggests that the neuroectodermal stem cells that were grafted could prevent secondary spinal cord damage and induce regeneration [31].

More recently, human HC016 (hHC016) cells, derived from human adipose mesenchymal stem cells (hAMSCs), were used in an acute SCI model. Functional recovery up to almost 2 months post-injury was assessed using locomotor (with CW) and sensory tests. The functional results showed a significant improvement on locomotor recovery with an enhanced survival capacity of hHC016 cells [32].

In the same year, another group used neural precursor cells (NPCs) for neuroregeneration in an SCI. To overcome the low survival rates of these cells in the injured sites, growth factors were added. A “cocktail” of EGF, bFGF, and PDGF-AA was identified as differentiation molecular sustainers for oligodendroglial and neuronal lineage differentiation. In a Wistar rat SCI model, this transplantation therapy was tested. Besides histological evaluation, the animals had significantly improved parameters evaluated on CW in terms of “stride length” and “average speed” 8 weeks after SCI [33]. The same group had previously shown that NPC transplantation reduces the acute and sub-acute inflammatory response after an SCI, attenuating chronic inflammation and improving neuroregeneration [34].

Mesenchymal stem cell (MSC) transplantation was tested in Lewis adult female rats with an SCI experimental model. Almost a month after the injury, a walking track test using CW has shown that gait recovery is significantly improved in MSC-transplanted animals [35].

Besides cell transplantation, various compounds were tested. A recent study evaluated a new treatment based on the removal of glutamate. Glutamate as a lesion enhancer is involved in excitotoxicity, inflammation, scarring, and axonal degeneration after an SCI. Therefore, blood glutamate scavengers (BGSs), recombinant enzyme glutamate-oxaloacetate transaminase (rGOT1), and its co-substrate oxaloacetic acid (OxAc) were tested in a SCI murine model. Besides various tests, locomotor function was assessed for 3 months using the CW system. BGSs have a proven good neuroprotective effect by reducing excitotoxicity and secondary damage following an SCI [36].

An SCI induces fibrotic scar formation and the generation of inhibitory factors, restricting the growth potential of injured neurons, as Sandner et al. showed [37]. In a female Fischer 344 rats SCI model, the epothilone-D therapy showed a functional benefit when analyzed in the Catwalk gait system [37].

Other therapy approaches were tested, such as simultaneous and combined early locomotor treadmill training (Tm) and injury site magnetic stimulation (TMSsc). In a rat model of a moderate SCI, it was shown that combined therapy (TMSTm) significantly induced lower spasticity, significantly more normal limb coordination (in CW gait analyses), and significantly greater forelimb grip strength. At the injury site, the TMSTm-treated animals had smaller cavity volumes and a better preservation of the white matter. Moreover, all the molecules involved in regeneration (e.g., dopamine beta-hydroxylase, glutamic acid decarboxylase, gamma-aminobutyric acid receptor B, brain-derived neurotrophic factor) were up-regulated [38].

The linear combination of several CW parameters into a SCI gait index score can better assess the gait recovery in experiments [22]. As it was shown in Section 2 of this review, Timotius et al. developed a linear discriminant analysis (LDA) method for evaluating a severe SCI in rats at the thoracic level. This method reflects the integrity/dysfunction of walking regardless of sex, strain, and weight. The P_LDA_ (parameters based upon LDA analysis) also evaluates the improving gait function after treatment. The proposed system of analysis with a higher sensitivity than others, integrates multidimensional parameters defining the quality of locomotion such as spatial, temporal, interlimb, and variation in interlimb coordination [22].

A disease that is related to an SCI is degenerative cervical myelopathy (DCM), which leads to chronic compression of the spinal cord. In an animal model of DCM, the efficacy of perioperative methylprednisolone (MP) was tested. The locomotor outcomes were registered using the CW system, and it was shown that MP reduces perioperative neurological complications following decompressive surgery [39].

### 3.2. Peripheral Nerve Injury

The peripheral nervous system is also subjected to injuries and affects around 3% of all trauma patients. Therefore, such as in SCI, there is a constant search for new regeneration therapies and procedures. The pre-clinical models aiming to test these new approaches are seminal in the translation to human applications. Computerized gait analysis permits a comprehensive acquisition of locomotor function. CW gait analysis can assess in animal models the peripheral nerve injuries of the upper and lower extremities, for example axonotmesis, neurotmesis, or fibrosis. Recently reviewed by Heinzel et al., this technology allows designed and comparable studies for the accelerated development of therapies in peripheral repair and regeneration [17].

Such as in an SCI, neuro-transplantation and cell therapies are the most common procedures tested in neuro-regeneration.

Autologous nerve transplantation was tested with an aligned chitosan fiber hydrogel (ACG) loaded with a bioactive peptide mixture to repair a sciatic nerve defect. The peptides used were mimicking the brain-derived neurotrophic factor (BDNF) and vascular endothelial growth factor (VEGF). The complex compound ACG-RGI/KLT was tested in vitro and further in an in vivo animal model. ACG-RGI/KLT oriented the Schwann cells and induced both the proliferation and secretion of neurotrophic factors. After 3 months, the nerve regeneration and the functional recovery in rats were registered using the CW system [40].

Surgical procedures are another an approach to regenerate peripheral nerves, but details in this procedure can have a tremendous impact on the process itself. Therefore, in ischiatic nerve injury performed in Wistar rats, heterologous fibrin sealant associated with a smaller number of stitches was tested. Besides the various testing approaches, CW analysis was used to test this improved surgical procedure. The analysis showed that the stitches reduction and heterologous fibrin sealant greatly minimizes the trauma and enhances the nerve reconstruction [41].

Sciatic nerve injury (SNI) is the most common model for investigating new therapies and approaches in peripheral nerve injury. This damage inflicts lesions to both motor and sensory fibers. Using transplanted cells, human muscle progenitor cells (hMPCs) that were genetically modified to overexpress neurotrophic factor (NTF) genes promoting axon regeneration and functional recovery, an SNI mouse model was developed. As soon as one week after SNI and following cell transplantation, CW registered parameters were found to be improved. The study shows the feasibility of future clinical application in humans’ SNI [42].

To improve transplantation and reduce rejection, in a similar mouse model of SCI, human amniotic mesenchymal stem cells overexpressing TGF-β were tested. Using various tests and CW assessment, it was shown that the stem cells overexpressing TGF-β induce rejection inhibition after xenotransplantation [43].

Fang et al. [44] showed that small gap tubulization after nerve transection is a promising approach for the in vivo functional recovery of a rat’s sciatic nerve. In this model, a reduced grapheme oxide-based conductive conduit was tested. The results of CW analysis suggested that an electrically conductive conduit promoted sensory and motor nerve regeneration and multiple-bud regeneration. Several landmarks of the footprints were considered, such as stand, max contact area, swing, duty cycle, and relative paw position. The rats’ correct walking depends on a coordination between sensory input, motor response, and cortical integration, and the track analysis can evaluate the recovery of nerve function during regeneration. The animal tends to place the hind paw at the previous position of the forepaw and the discrepancy of footprints and footfall patterns between animal groups with nerve transection, nerve transection with small gap tubulization, and controls is one of the mandatory tests made with CW [44].

Human embryonic stem cells (hESCs) were tested in an SNI mouse model in combination with a heterologous fibrin sealant scaffold. The recovery rate was tested with CW and it was demonstrated that the hESCs engineered to overexpress FGF-2 can support neuro-regeneration at both the motor and sensory functions level [45].

Other types of therapies were tested in SNI analogue with the ones tested in SCI models. The animals with SNI were tested for an approach using high-frequency transcutaneous neuromuscular electrical nerve stimulation (TENS) treatment. The CW gait analysis showed that immediate electrical stimulation induced a significantly high regularity index [46].

Although the main model for peripheral nerve regeneration is the rat SNI model, another animal model gains importance. The femoral nerve model does not inflict auto-mutilation in experimental animals. In Sprague Dawley rats, after the resection of the right femoral nerve and reconstruction using an autologous nerve graft, 10 weeks post-surgery, several parameters were investigated (e.g., Print Area, Print Length, Swing Speed, Duty Cycle). Two weeks after surgery, all the parameters were decreased, and after 10 weeks all the parameters recovered to more than 90% in comparison to the baseline. Moreover, authors emphasize on the differences between the models of femoral nerve and SNI [47].

Human dental pulp-derived cells (DPCs) transplanted for peripheral nerve regeneration after crushing in a SNI rat model were also studied. Fourteen days after surgery, the DPCs transplanted animals proved, on the CW system, a recovery of motor function by a higher ratio of tibialis to anterior muscle weight. The study showed a good correlation between the morphological and functional findings after DPC transplantation regarding peripheral nerve regeneration [48].

Human neural crest stem cells (hNCSCs) were also tested in SNI models. Du et al. used CW gait analysis, along with other methods, such as the gastrocnemius muscle index, electrophysiology, immunohistochemistry, and histomorphometric analysis, to evaluate the rat gastrocnemius muscle after sciatic nerve injury and hNCSCs transplantation. They showed that hNCSCs at the fifth passage having a much higher therapeutic efficacy than those at the sixth passage is important in terms of the regeneration potency, the manipulation of hNCSCs, and the time of transplantation [49].

Various diseases that are associated with peripheral nerve destruction were also tested in animal models in the quest to search for new therapies and approaches. Peripheral neuropathy is a common complication of diabetes associated with pain and motor function decline. In Lewis rats, diabetes was experimentally induced and, in the CW system, mechanical hyperalgesia and a reduction in the hind limbs footprint intensities was investigated. Alterations in the spatial parameters (e.g., Maximum Contact Area; Stride Length; Print Area) were correlated with the mechanical withdrawal thresholds. Authors point out in this recent study that the CW gait parameters are a good complementary tool to investigate the development of hyperalgesia diabetic neuropathy [50].

The CW system, developed some ten years ago, continues to be an important tool in the pre-clinical research due to its reliable and detailed analysis of gait in various disease rodent models.

## 4. Discussion Regarding General Outlines Using Computerized Gait Analysis System and its Effectiveness in In Vivo Experiments

In humans, minor speed changes (approximately 0.1 m/s) are predictive biomarkers of various neurological disorders, such as hemiplegic stroke [51], inflammatory CNS diseases, peripheral neuropathies, vertigo syndromes, cerebrovascular CNS diseases, and idiopathic normal pressure hydrocephalus [52]; therefore, systems that can objectively evaluate various gait characteristics in animal models are extremely useful.

CW is one of the very useful systems, developed in recent years, which can objectively evaluate the walk of rodents in various in vivo models of nerve recovery after injury. It is known that the experimental in vivo models for assessing the evolution of peripheral nerve and SCI before and after treatment provide valuable data regarding the methods of therapeutic approaches in humans. Translational medicine shows that the safer, more objective, more accurate, and faster the evaluation methods are, the faster the translation of data from the research bench to the patient’s bed is.

Rodents are used as the primary pre-clinical model for many diseases and the gait automated system can adequately measure a multitude of gait parameters such as static parameters (base of support, stride length, maximum contact area, relative paw position), dynamic parameters (stance, swing, and step cycle durations) and coordination parameters (regular step patterns, regularity index, phase legs, locomotor speed) [53]. The animal models mimicking human neurological disorders represent the basic research avenue for both studies of disease mechanisms and of improved therapies/drugs.

Damages can occur in the central and peripheral nervous systems (e.g., stroke, trauma, tumor, degenerative neurologic disorders, or substance abuse) [54]. Although rodents are not ideal models for some human diseases, they are sometimes the only preclinical models, genetically perfectible, to provide valuable preclinical experimental data.

Our purpose in this review was to analyze the results obtained in the last few years regarding the evaluation of walking parameters in spinal cord and peripheral nerve recovery after injury using CW as an objective and reliable method.

There are several methods to quantify locomotor behavior, such as electrode recordings, footprint analysis, 2D and 3D techniques for the determination of limb kinematics, visual observation of animals moving in an open field, and Gait analyzing systems [14]. The latter does not replace all the others, but complement them and bring valuable evaluation data, as evidenced by the numerous studies in which it has been used. CW for rodents has proved to have many advantages, such as the speed of locomotion control and the automated data acquisition and processing. The evaluation of static and dynamic gait parameters can be performed in a variety of neurological animal models, both in central and peripheral nerve injury [5,6,7]. The study of Heinzel et al. on rats with an autograft after the resection of the median nerve also showed that CW automated gait analysis is a feasible tool for assessing functional recovery in these animals. Given the complexity of quadrupedal locomotion, the advantage of this method of analysis compared to the isolated flexion of the digits becomes obvious. The CW test showed that it correlates well with other assessment methods such as grasping strength measurements and electrophysiology [18]

As Kappos points out, the parameters assessed using the CW can be divided into “general parameters”, “qualitative data”, and “quantitative data”, that can be used for statistical analysis [14]. Quantitative data include static and dynamic paw parameters, and coordination parameters. The authors showed that the assessment has been used mostly for nerve injury [5], arthritis [55], modelling pain [50], but also in a few studies for neurodegenerative diseases that affect the gait [15]. The system was developed to evaluate footfall and gait changes, and it can calculate 25 parameters based on footprints and 10 parameters based on time interval. It provides information for each run about paw positions, paw print sizes, and paw intensities as a function of time or video frame [56].

Crowley et al. revealed that in animal models of SCI, the system can provide an unbiased quantitative assessment of the refined aspects of locomotor function and, therefore, it can detect significant differences between pre-injury and post-injury data. As stated several times in our paper, human errors and subjectivity are significantly reduced when using CW. The data obtained by taking high-resolution videos of the animals walking, analyzed with a specialized software, could be combined, creating a simple mathematical model, the Combined CatWalk Index (CCI) [5]. This study showed that the CCI system produced similar results to the Basso Mouse Scale (BMS) or the CW’s Step Sequence Regularity Index, but with a significant smaller coefficient of variation. Moreover, CCI scoring showed a slightly better correlation with impact force. CCI correlated 104 CW parameters and BMS data using linear regression. All the linear regression equations were then combined into a single weighted average. The weighting factor, R^2^, was used to determine parameters with strong correlation (with strong weights). The BMS method has the advantage of a single score that could be easily compared between individual mice. Nevertheless, training is required before using this method. The CW system has the advantage of a greater objectivity but choosing the right parameters for making a comparison between mice could cause problems. Thus, a combined CW index using the advantages of both systems, CCI and BMS, seemed to be a better choice. However, a significant limitation may be the linear regression model used in this work. Moreover, a correlation with spinal cord damage using histological studies was not performed. However, the authors suggested that CCI is likely to predict spinal cord tissue damage.

A recent study regarding the SCI in rodents has shown that many system’s outcome variables are highly correlated and dependent on run speed. The CW system has shown to detect significant differences between experimental groups of animals compared with the control ones. However, the summary group level data could obscure the variability between each subject of the experiment; therefore, there is a difficulty in understanding the magnitude of effect in individual rodents. In this situation, calculating reference change intervals (RCIs) is important in an experimental design for quantifying variability and providing details of individual level change. RCIs define the limits of normal variability for the values of rats locomotion scales on CW, thereby differences up to 70% from the baseline value must be considered normal [16]. As the multitude of variables that CW offers to quantify the results of an experimental model of SCI, it is necessary to select the variables that define the efficacy of spinal cord intervention before the experiment begins, not restricting analysis to run speed or duration. It was suggested to use, in SCI studies, stride length, swing duration, base of support, and duty cycle as suitable measures for hind limb use. To quantify coordination, an outcome compulsory to follow, is hind limb–forelimb coupling.

Guo et al. showed that in an SCI performed by contusion or distraction, walking deficits and recovery could be affected by the type of traumatic SCI, and they used CW to perform the gait analysis of animals walking. The CW parameters for interpaw coordination (step sequence patterns and duration), paw support (base of support, print position), and front and hind paw movements (paw maximum contact area time, pressure applied to the floor, the stand time and duty cycle, paw angle, swing duration, paw stride length) were tested together with the skilled locomotor movements of animals walking across the ladder rung walking test apparatus. The two test results demonstrated their complementarity in determining the differences between the two spinal cord injury models [29].

Bozkurt et al. used a rat sciatic nerve crush injury model in the CW analysis of behavioral recovery after twelve weeks [20]. The group found several parameters of interest for the detection of nerve recovery, such as dynamic gait variables, coordination measures, and the intensity of paw prints, and they concluded that the system simultaneously demonstrated both static and dynamic gait parameters.

Some improvements of data presentations have also been reported for CW analysis. Hence, in order to avoid arbitrary parameter selection, heat mapping of the initial data analysis was shown to be advantageous in reporting clustered gait parameter differences [15]. Moreover, a report in 2018 on the application of machine learning in CW analysis has shown that various parameters (step decomposition, the definition and extraction of meaningful features, multivariate step sequence alignment, and feature selection, among others) could be “learned”. This was the first report on the ability of machine learning to pharmacologically discriminate relevant groups based on their walking behavior [19].

An evaluation of the efficacy of neurological disease treatment in in vivo models has been commonly conducted through the application of neurobehavioral tests. However, despite the attempts of numerous studies, the results cannot be applied to humans. In the opinion of Vidal et al., this discrepancy is due to poor standardization in the behavioral protocols and their execution among investigators [57]. The correlation between body weight, speed, and system parameters should be calculated. For example, if an experiment lasts weeks or months, the animals’ ages and the speed of movement can be physiologically influenced not only by the protocol of the experiment but also by aging. The same can be said for body weight, a parameter known to transiently drop after injury. Many CW parameters are dependent on these two variables; therefore, instead of comparing one or two time-points using a classical *t*-test or one-way and two-way ANOVA, it would be better to use models such as a linear mixed model (LMM) or linear mixed-effects (LME) model for a long-term characterization by CW [39].

Another issue regarding the evaluated parameters is the standardization between laboratories. It was reported that CW could evaluate rodent motor function using specialized software that permits the diminution of inaccuracies due to human error and subjective interpretations. However, it is not always clear what data are important for the study purpose. Crowley et al. has shown that a mathematical model can be created using data collected during a mouse SCI experiment, named the Combined Catwalk Index (CCI). The CW software produces a large amount of data, with a total of approximately 104 parameters [5]. Hence, the investigator must choose those parameters that are relevant and adequate to each study. The software may not be perfect but can potentially be perfected through researchers’ optimization.

## 5. Conclusions

The translational success of therapies and approaches in nerve regeneration relies on testing using animal models. Spinal cord and peripheral nerve injuries are neuropathologies inducing massive physical and emotional distress as well as irreversible disabilities, for which significant social and economic burdens may be implicated. Spinal cord and peripheral nerve injuries are severe deficits that result in heavily impaired motor execution in humans. Unfortunately, no perfect curative treatment is available in both types of mechanical injuries and/or in diseases that lead to these impairments. Therefore, intensive efforts involving both in vitro and in vivo experimental models are being made to test new drugs, new therapies, and new therapeutic approaches. As part of this quest, important analysis tools, such as automatic gait analysis, are seminal in providing results that can quickly be translated into human application.

To achieve proper analysis in these investigations, an approach involving a combination of electrophysiological tests, histological and immunohistochemical studies, and behavioral exploration is recommended. One of the important behavioral testing paradigms is the CW system, whose application has been demonstrated using both static and dynamic gait parameters, with a great impact in the evaluation of nerve regeneration in the central or peripheral nervous system. The CW method offers an appropriate quantitative analysis of locomotion in small animals, allowing for the quantification of an extensive number of gait parameters.

## Figures and Tables

**Table 1:** The frequently used CW parameters and the references in which they were reported.

Category of Parameters	Name of Parameters	Description	References
Dynamic paw parameters	Stance duration	Duration of the stance phase.	[13,14,15,16]
	Stand duration	Stand time of the paw; stand time/stance phase (sec).	[17,18]
	Swing duration	Duration of the step cycle phase; swing time/swing phase.	[13,14,15,16,17,18]
	Step cycle duration	Duration of the step cycle (stance and swing duration) (sec).	[14]
	Duty cycle (%)	Stand time divided by stand time + swing time.	[15,16,17,18]
Static paw parameters	Base-of-support (BoS)	The distance between the two forelimbs or between the two hind limbs.	[13,14,16,17,18]
	Stride length	Length (mm) of a stride; distance between two consecutive placements of a paw.	[13,14,16,17,18]
	Maximum contact or print area	The total floor area contacted by the paw during the stance phase; expressed in square pixel.	[13,14,16,17]
	Minimum paw print intensity	Minimum intensity of the paw.	[17,19]
	Mean paw print intensity	Mean intensity of the paw.	[13,16,17,18,19]
	Maximum intensity at maximum contact	Maximum intensity of the paw at maximum contact.	[17,19]
	Relative paw position	Relative positions of fore and hind paws: hind paw position is related to the previous forepaw position. If the hind paw is placed (partially) after the forepaw, the distance is positive; otherwise, it is negative.	[14]
	Print length	Length of paw print.	[13,17,18,19]
	Print width	Width of paw print.	[13,15,17,18,19]
	Print area	Area of paw print.	[13,16,17,18,19]
Coordination parameters	Regular step patterns	The definition of regular step patterns is based upon walking: during the (faster) trot, diagonal pairs are placed almost simultaneously. If two paws become visible at the same time, the program assigns precedence to the paw that occludes the largest area at that moment.	[14]
	Normal step sequence patterns (NSSP)	Specific sequences of paw placements during a step cycle.	[17,18]
	Phase dispersions/phase lags/couplings (%)	Temporal differences between the step cycles of two paws.	[16,17,18]
	Regularity index (RI)	The RI defines interlimb coordination as the exclusive use of regular step patterns during uninterrupted locomotion.	[13,14,15,17]
	Phase lags	Another measure for interlimb coordination based on time relationships between footfalls; the moment of initial contact of one paw is related to the stride cycle of another paw.	[13,14]
	Locomotor speed (average speed)	The average speed of walkway crossing (cm/sec) was calculated automatically by dividing the covered distance (cm) of the walkway through the time (sec) needed to cross it.	[14,16]
Functional indices (for sciatic nerve experiments)	Sciatic functional index	Index including print length on both the experimental and the normal sides, toe spread between the first and fifth digits on both sides, and the distance between the middle of the second and the fourth toes on both sides.	[14]
	Static sciatic index	Index containing the ratios of hind foot parameters (1–5 toe spread factor (TSF) and intermediate toe spread factor (ITF)) of both injured and uninjured paws.	[14]
Other	Swing speed (cm/sec)	Swing speed of the paw.	[17,18], [15,16]
	Stand index (%)	Measurement for the speed with which the paw loses contact with the ground.	[15,17,18]
	Single stance/Single stand (sec)	Time when stance is maintained by the respective paw only.	[17]
	Step cycle (sec)	Duration of stance phase + swing phase of the paw.	[17]
	Paw placements (PP)	Number of placements of the respective paw.	[17]
	Run duration (sec)	Duration of the walkway crossing.	[16,17]

## Data Availability

The data presented in this study are available on request from the corresponding author.
